# A bispecific antibody AP203 targeting PD-L1 and CD137 exerts potent antitumor activity without toxicity

**DOI:** 10.1186/s12967-023-04193-5

**Published:** 2023-05-25

**Authors:** Po-Lin Huang, Hung-Tsai Kan, Ching-Hsuan Hsu, Hsin-Ta Hsieh, Wan-Chien Cheng, Ren-Yeong Huang, Jhong-Jhe You

**Affiliations:** 1 AP Biosciences, Inc., 17F., No. 3, Yuanqu St., Nangang Dist., Taipei, 115603 Taiwan; 2 Department of Periodontology, School of Dentistry, Tri-Service General Hospital and National Defense Medical Center, Taipei, Taiwan

**Keywords:** Bispecific antibody, PD-L1, CD137, Cancer immunotherapy, Antitumor immunity

## Abstract

**Background:**

Bispecific antibody has garnered considerable attention in the recent years due to its impressive preliminary efficacy in hematological malignancies. For solid tumors, however, the main hindrance is the suppressive tumor microenvironment, which effectively impedes the activation of infiltrating T cells. Herein, we designed a bispecific antibody AP203 with high binding affinity to PD-L1 and CD137 and assessed its safety and anti-tumor efficacy, as well as explored the mechanism of action.

**Methods:**

The optimal antibody binders against PD-L1 and CD137 were screened from the OmniMab phagemid library. The binding affinity of the constructed AP203 were evaluated using enzyme-linked immunosorbent assay (ELISA) and biolayer interferometry (BLI). T-cell stimulatory capacity was assessed using the allogeneic mixed lymphocyte reaction (MLR), antigen-specific recall response, and coculture with PD-L1-expressing cells. In vivo antitumor efficacy was evaluated using two models of tumor-xenografted humanized mice with profiling of tumor infiltrating lymphocytes (TILs). The possible toxicity of AP203 was examined using in vitro cytokine release assay by human PBMCs.

**Results:**

AP203, which simultaneously targeted PD-L1 and costimulatory CD137, elicit superior agonistic effects over parental antibodies alone or in combination in terms of T cell activation, enhanced memory recall responses, and overcoming Treg-mediated immunosuppression (P < 0.05). The agonistic activity of AP203 was further demonstrated PD-L1-dependent by coculturing T cells with PD-L1-expressing cells. In vivo animal studies using immunodeficient or immunocompetent mice both showed a dose-related antitumor efficacy superior to parental antibodies in combination (P < 0.05). Correspondingly, AP203 significantly increased tumor infiltrating CD8 + T cells, while decreased CD4 + T cells, as well as Treg cells (P < 0.05), resulting in a dose-dependent increase in the CD8 + /CD4 + ratio. Moreover, either soluble or immobilized AP203 did not induce the production of inflammatory cytokines by human PBMCs.

**Conclusions:**

AP203 exerts potent antitumor activity not only by blocking PD-1/PD-L1 inhibitory signaling, but also by activating CD137 costimulatory signaling in effector T cells that consequently counteracts Treg-mediated immunosuppression. Based on promising preclinical results, AP203 should be a suitable candidate for clinical treatment of solid tumors.

**Supplementary Information:**

The online version contains supplementary material available at 10.1186/s12967-023-04193-5.

## Introduction

Tumor immune surveillance is an important protective mechanism by which the immune system monitors, recognizes and eliminates precancerous and/or cancerous cells in the body [1]. Nonetheless, tumors exploit cancer immunoediting and immune-tolerance mechanisms to escape immune surveillance and thus continue tumor development in the presence of a functioning immune system [2, 3]. As a result, immune checkpoint blockade-based immunotherapy has gained significant attention in the last decade, with the two most prominent immune checkpoints being the programmed cell death-1 (PD-1)/programmed cell death ligand-1 (PD-L1) axis and the CD137/4-1BB costimulatory axis [4–6].

PD-1/PD-L1 blockade plays an important role in immune tolerance, which is critical for tumors that escape cancer immunosurveillance and subsequent elimination [7]. PD-1 is mainly expressed on the cell surface of effector T (Teff) cells, whereas PD-L1 is constitutively expressed on the surface of various tumor cells. Engagement of PD-1 and PD-L1 triggers immune tolerance by promoting Teff cell suppression and regulatory T (Treg) cell maintenance, which contributes to increased tumor cell survival [8]. Therefore, therapeutic antibodies than block PD-1/PD-L1 engagement can restore anti-tumor immunity to control and eliminate tumors [9–12]. Despite the initial success in various tumors, a considerable portion of patients do not response to therapeutic PD-1/PD-L1 antibodies and are associated with poor survival outcomes [13–15]. Therefore, different combinations of checkpoint blocking antibodies such as CTLA-4 have also been tried and indeed greatly improved response rates and survival benefits [16, 17]. On the opposite end of the spectrum, however, the strategy of combined checkpoint blockade is believed to be associated with an increased risk of immune-related adverse events (irAEs) in clinical practices [18–22]. Therefore, in addition to combining other suitable immune checkpoint blockades, breakthrough immunotherapies, such as T cell-dependent bispecific antibody, are needed.

CD137 (4-1BB/TNFRSF9), an inducible costimulatory receptor, has recently received increasing attention as a therapeutic target for cancer therapy [6, 23]. CD137 is mainly expressed on the surface of activated T cells, dendritic cells (DCs), and natural killer (NK) cells [24]. Upon engaging with its trimeric ligand CD137L or crosslinking with agonist antibodies, activated CD137 can provide costimulatory signals to promote T cell proliferation, survival, and effector function, as well as enhance secretion of IL-2 and IFN-γ cytokines [25]. In different preclinical tumor models, CD137 agonistic antibody have been shown to effectively elicit potent anti-tumor immune responses [6, 26, 27]. In addition to monotherapy efficacy, combination of CD137 agonistic antibody and PD-1/PD-L1 blocking antibodies can synergistically enhance anti-tumor effects, reduce Treg cell infiltration, and prolong survival [27–31].

In addition to the combination strategy of standard antibodies, bispecific antibodies provide a variety of potential functional advantages, such as superior cytotoxic effects and lower rate of resistance, due to their highly matched targeting to two different antigens simultaneously [32]. Currently, bispecific antibodies have achieved excellent clinical efficacy the treatment of hematological malignancies, such as acute myeloid leukemia, lymphoma, myeloma, and non-Hodgkin's lymphoma [33–36]. For solid tumors, which account for 90% of all cancer, however, the current progress of bispecific antibody is still in the development and clinical evaluation [37, 38]. The main challenge of solid tumors is immunodeficiency caused by suppressive tumor microenvironment. Based on encouraging results from preclinical studies and clinical trials, PD-1 and CD137 emerge as potent candidates for bispecific antibody binders to overcome immunosuppressive tumor microenvironment. In this study, we developed an engineered bispecific antibody AP203 that mounted a superior CD137 agonistic activity upon PD-L1 engagement for T cell activation. Through this PD-L1-dependent T cell activation, AP203 provides a better benefit-risk profile to support clinical development.

## Materials and methods

### Cell lines and reagents

The human MDA-MB-231, NCI-H292, PC-3, BxPC-3, and NCI-H1975 cell lines were used as PD-L1 positive targets, which were all purchased from Bioresource Collection and Research Center (BCRC, Hsinchu, Taiwan). The HEK293-F cell line was purchased from Thermo Fisher (Thermo Fisher Scientific). All cell lines were cultured following instructions of suppliers. Anti-HEL (Hen Egg Lysozyme) human IgG1 isotype was in-house generated and used as negative control. The human peripheral blood mononuclear cells (PBMCs) were collected from healthy donors with ethical approval by Tri-service General Hospital National Defense Medical Center, Taiwan (#1-107-05-193).

### Construction of bispecific antibody AP203 targeting PD-L1 and CD137

The OmniMab phagemid library (AP Biosciences), constructed from IgG sequences of healthy donors’ PBMCs, was used to isolate and select specific binders for PD-L1 and CD137. In the first-round screening, antibody phage display was prepared using the Hyperphage M13 (KO7ΔpIII, Progen, Heidelberg, Germany). Solid phase planning and cell panning were used to identify positive phage clones binding to PD-L1 or CD137. Homemade recombinant human PD-L1 and extracellular domain of CD137 fused to the N-terminus of the Fc region of IgG (CD137-ECD-Fc) was used in the first-round solid phase panning, while HEK293 cells expressing PD-L1 or CD137 were used for the second and third rounds of enrichment. After three rounds of panning, positive phage clones binding to PD-L1 or CD137 were screened and isolated by enzyme linked immunosorbent assay (ELISA) and fluorescence-activated cell sorting (FACS) analysis. The heavy chain variable domain (VH) of these positive phage clones derived from the OmniMab phagemid library were sequenced. All unique clones were cloned into an IgG backbone vector, and the antibody binders were produced in transiently transfected ExpiCHO cells (Thermo Fisher Scientific) for subsequent binding affinity screening. Bispecific antibody, named AP203, were then generated using the two potent lead binders for PD-L1 (clone #6) and CD137 (clone #54) with a flexible (GGGGS)_2_ linker. Two single-chain variable fragment (scFv) targeting CD137 were fused to the C-terminus of the heavy chain of a PD-L1 antibody (IgG1 format) with a short flexible (GGGGS)_2_ linker (Additional file 1: Figure S1A). To diminish N-glycosylation and antibody-induced allodynia, N297A and K322A mutations were generated on the Fc region of AP203 by site-directed mutagenesis. The constructed bispecific antibody AP203 was expressed using the Gibco ExpiCHO Expression system according to the manufacturer’s instructions. The bispecific antibody AP203 was then purified by Protein A-affinity chromatography. The purity of AP203 was analyzed by size-exclusion chromatography-high performance liquid chromatography (SEC-HPLC), and purity greater than 95% were combined for subsequent analysis (Additional file 1: Figure S1B).

### PD-L1 and CD137 binding activity by direct ELISA

Human PD-L1 or CD137 IgG1 Fc chimera was coated on Nunc Maxisorp 96-well plates (Thermo Fisher Scientific) overnight at 4 °C. The wells were washed with 1X wash buffer (0.1% Tween-20 in PBS) to remove unbound antigen and then blocked with PBS containing 5% low-fat milk for 1 h at room temperature with orbital shaking. After washing, serially diluted PD-L1 or CD137 antibodies was added and incubated for 1 h at room temperature. The bound antibody/antigen complexes were detected by incubation with horseradish peroxidase-conjugated goat anti-human IgG, F(ab’)2 specific F(ab’)2 antibody (1:2000 dilution; Jackson Immunoresearch #109-036-097) for 1 h at room temperature and then revealed by incubation with TMB substrate (Invitrogen). The reaction was stopped by adding an equal amount of 1N HCL, and absorbance was then measured at 450 nm using a spectrophotometer (Bio-Tek Spectra).

### PD-1/PD-L1 blockade assay

Recombinant human PD-1-His protein was prepared in-house and coated (250 ng/well) on Nunc Maxisorp 96-well plates (Thermo Fisher Scientific) overnight at 4 °C. After removing unbound PD-1 protein, the wells were blocked with 5% low-fat milk in PBS. Serially diluted PD-L1 antibodies (from 200 to 0 nM) were premixed with an equal volume of recombinant PD-L1-Fc chimeric protein (75.5 nM) and added to the PD-1-coated plates with 1-h incubation at room temperature. The plate were washed again, and PD-L1 antibodies bound to the coated PD-1-His protein were detected using HRP-conjugated goat anti-human IgG. The competition ability of the PD-L1 antibody was monitored by adding TMB (Invitrogen) and measuring absorbance using a spectrophotometer (Bio-Tek Spectra).

### Analysis of biolayer interferometry (BLI)

The simultaneous binding of AP203 to PD-L1 and CD137 antigens was determined by the Octet RED96e system (FortéBio, USA). Briefly, His-tagged human PD-L1 antigen (5 μg/ml) was first loaded onto a HIS1K biosensor (Anti-Penta-HIS, ForteBio, Cat#18-5120) and then used to capture AP203 (100 nM, 5 min), followed by exposure to Fc-tagged human CD137 antigen or isotype control (100 nM, 5 min) to check the additive signal caused by dual-binding.

### Analysis of agonist activity of screened anti-CD137 antibodies

Human T cells were isolated from the blood of healthy adult volunteers using RosetteSepTM Human T Cell Enrichment Cocktail (STEMCELL, #15061). Isolated human T cells (1 × 10^5^ cells) were inoculated onto 96-well Nunc MaxiSorp^™^ plates precoated with anti-CD3 antibody (OKT3, Biolegend, 1 μg/mL) and screened anti-CD137 antibodies. After 3 days of incubation, secreted IL-2 and IFN-γ levels were determined by ELISA assay.

### Mixed lymphocyte reaction (MLR)

Human CD14 + monocytes were isolated by RosetteSep™ Human Monocyte Enrichment Cocktail (STEMCELL; #15068) and cultured in RPMI-1640 medium (Gibco) supplemented with 10% FBS (Gibco). Human CD14 + monocytes were then differentiated into immature dendritic cells (iDCs) by stimulation with human GM-CSF (1000 IU/ml; R&D) and IL-4 (1000 IU/ml; R&D) for 5 days. Allogenic CD4 + T cells were isolated by RosetteSep™ Human CD4 + T Cell Enrichment Cocktail (STEMCELL; #15062) and then labeled with CFSE (Thermo Fisher Scientific; # C34554). For MLR experiments, iDCs (1 × 10^4^ cells) and allogenic CD4+ T cells (1 × 10^5^ cells) were co-cultured in a 96-well plate and then incubated with antibodies at the indicated concentrations. After 2 and 5 days, the secreted IL-2 and IFN-γ in culture supernatant were measured by ELISA kits (IL-2: Thermo Fisher Scientific; IFN-γ: BioLegend).

### Regulatory T cell suppression assay

Regulatory T (Treg) cells were isolated from PBMCs by EasySep^™^ Human CD4 + CD127lowCD25 + Regulatory T Cell Isolation Kit (STEMCELL, #18063) according to manufacturer’s instructions. Isolated Treg cells were further expanded using the human Treg Expander Dynabeads^™^ (Gibco) for 3 weeks. For Treg suppression assays, effector T (Teff) cells (5 × 10^4^ cells) simultaneously isolated by the Treg isolation kit were labelled with CFSE and cultured with Treg cells at different Teff/Treg ratios in the presence of allogenic DCs (1 × 10^4^ cells) in a 96-well U-bottom plate. After 2 days of treatment with the indicated antibodies, IL-2 levels in culture media were measured using ELISA (Thermo Fisher Scientific). After 5 days of culture, the proliferation of Teff cells was determined and quantified by Attune^™^ NxT Flow Cytometer (Thermo Fisher Scientific). Teff cells proliferation suppression by Treg cells was calculated using following formula: 100—(% of CFSE^low^_with Treg_/% of CFSE^low^_without Treg_), as described previously [39].

### Co-culture of CD8 + T cells with PD-L1-expressing tumor cells

Human CD8 + T cells were isolated by positive selection as previous described. Purified CD8 + T cells were cocultured with MDA-MB-231, NCI-H292, PC-3, BxPC-3, or NCI-H1975 tumor cells at a ratio of 1:1. Co-cultured cells were treated with indicated antibodies for 3 days, and the culture supernatants were collected and analyzed for IFN-γ secretion by using IFN-γ ELISA kit (BioLegend) according to the manufacturer’s instructions.

### Analysis of cytokine release from PBMCs isolated from healthy donors

Human PBMCs were isolated from healthy volunteers using the Lymphoprep density gradient separation (STEMCELL, #07811). To improve the sensitivity of T cells to immunostimulation, isolated PBMCs with high cell density were pre-cultured in RPMI-1640 medium (Gibco) supplemented with 10% FBS (Gioco). Subsequently, pre-cultured PBMC were stimulated with soluble or immobilized isotype IgG (anti-hen egg lysozyme antibody, AP Biosciences), anti-CD3 antibody (OKT3, Biolegend), anti-CD28 antibody (TGN1412, ichorbio), and bispecific antibody AP203 for 24 h. For immobilized antibodies, the antibodies were coated in the Maxisorp plate overnight at 4 °C. The secreted cytokines in culture supernatant were determined by ProcartaPlex Immunoassays (Thermo Fisher Scientific) was used to measure the secreted cytokine in culture supernatant, including IL-1β, IL-2, IL-6, IL-8, IL-10, IL-13, IL-17A, TNF-α, and IFN-γ.

### In vivo mouse models

Animal experiments performed in this study were approved by the Institutional Animal Care and Use Committees and in accordance with the regulations of care and management of experimental animals. For the immunodeficient SCID/beige mouse model, BxPC-3 pancreatic tumor cells (3 × 10^6^ cells) and freshly isolated human PBMCs (1 × 10^6^ cells) were mixed with Matrigel in a 1:1 volume ratio and inoculated subcutaneously into the lower flank of SCID/beige mice (BioLASCO). For immunocompetent human PD-1/PD-L1/CD137 triple knock-in mouse model, human PD-L1-expressing MC-38 colon cancer cells (5 × 10^5^ cells) were subcutaneously inoculated into the lower flank of triple knock-in mice (BIOCYTOGEN). After tumor size reached approximately 100–150 mm^3^, the mice were randomly divided into PBS vehicle control group, #6 and #54 combination group, and three escalating doses of AP203 groups (0.13, 1.3 and 13 mg/kg). Subsequently, the mice were injected with indicated antibodies twice a week by intraperitoneal injection. Tumor size was measured twice a week by measuring the tumor length and width. Tumor volume (mm^3^) was calculated using following equation: length × width^2^ × 0.5. Herein, the length represents the long dimension of the tumor, and width indicates the short dimension of the tumor.

### Flow cytometry analysis of tumor infiltrating lymphocytes (TILs)

Tumors were dissociated into a single cell suspension using the Tumor Dissociation Kit and gentleMACS Octo Dissociator (Miltenyi Biotec) according to the manufacturer’s instructions. TILs were separated using the Lymphoprep density gradient separation (STEMCELL), and the human T cell populations were analyzed by flow cytometry based on the surface expression of human CD45, CD3, CD4, CD8, and Foxp3. Antibodies used for TILs analysis were purchased from Biolegend. TILs populations were determined using a Attune™ NxT Flow Cytometer (Thermo Fisher Scientific) and analyzed using FlowJo software.

### Statistical analysis

Statistical analyses were performed using the GraphPad Prism Software for Windows (version 8.0.1). Differences between groups were assessed using Student’s t-tests, and significant differences were marked in each figure as * < 0.05, ** < 0.01, and *** < 0.001.

## Results

### Generation of AP203, a bispecific antibody targeting human PD-L1 and CD137

**Fig. 1:**
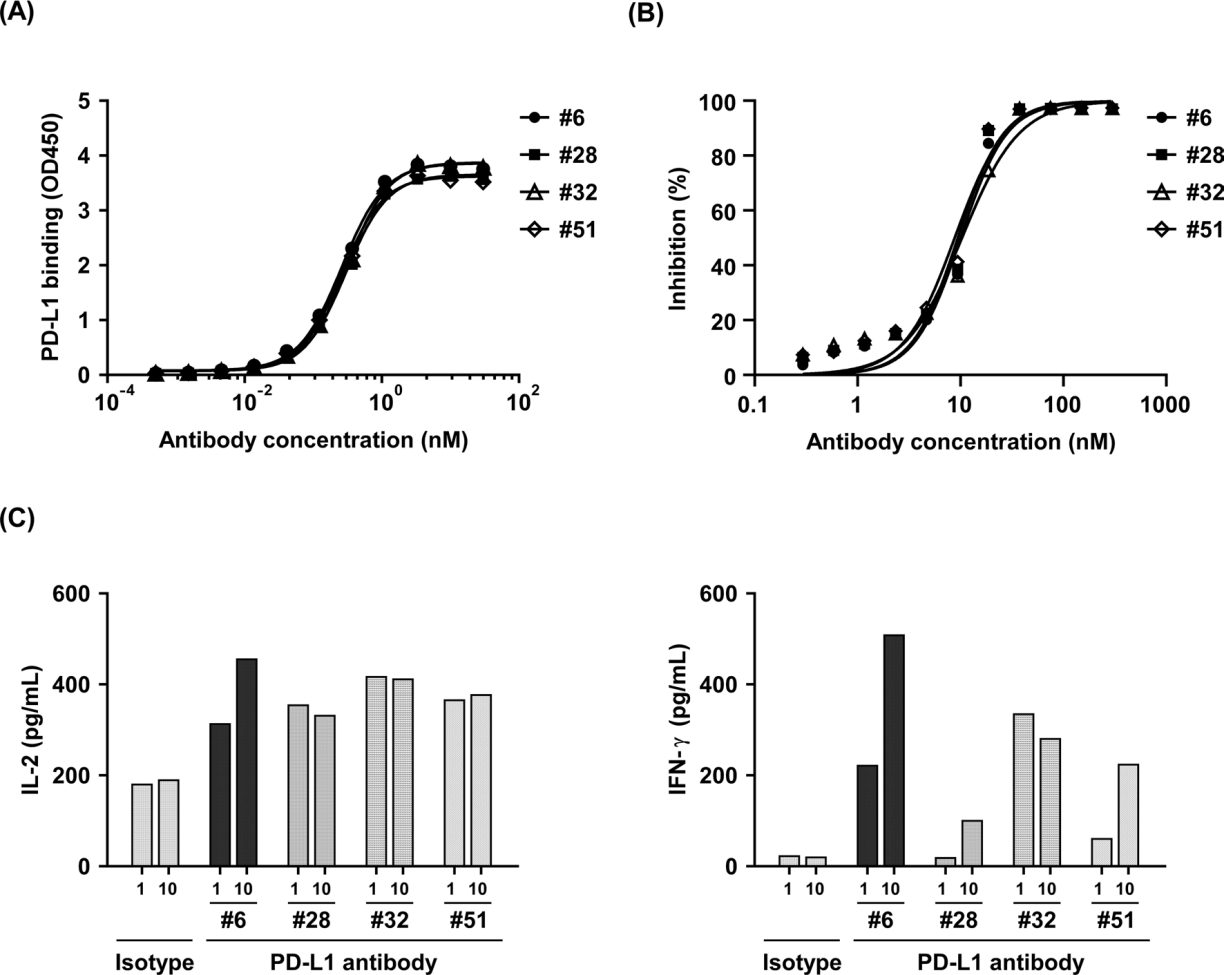
Screening of potent PD-L1 binders through OmniMab phagemid library. **A** Top four high-affinity binders that bind to PD-L1. The binding affinity of four PD-L1 antibody candidates to recombinant PD-L1 was determined using ELISA. **B** PD-1/PD-L1 blocking assay of the top four PD-L1 antibody candidates. Competitive binding of PD-1/PD-L1 was analyzed by ELISA. **C** The top four PD-L1 antibody candidates were analyzed for their ability to enhance CD4 + T cell activation in the allogeneic MLR. The secretion of IL-2 and IFN-γ in the culture supernatant was measured by ELISA. Clone #6 showed the better dose-dependent bioactivity in promoting the secretion of cytokine IL-2 and IFN-γ

Specific binder targeting PD-L1 were screened using the OmniMab phagemid library and then converted into IgG1 format. Figure 1A shows the top four clones with high binding affinity to fully human PD-L1, with an EC_50_ ranging from 0.26 nM to 0.31 nM. PD-1/PD-L1 blockage bioassay revealed that the four PD-L1 binders exhibited similar competitive binding activity against PD-1/PD-L1 interaction, with an IC_50_ ranging from 8.8 nM to 10.3 nM (Fig. 1B). Subsequently, the ability of these four PD-L1 binders to promote T cell response was assessed using an allogeneic MLR. As shown in Fig. 1C, PD-L1 clone #6 exhibited the better dose-dependent bioactivity than the other three clones in promoting the secretion of cytokine IL-2 and IFN-γ, suggesting that clone #6 is the potent anatgonist against PD-L1.

**Fig. 2:**
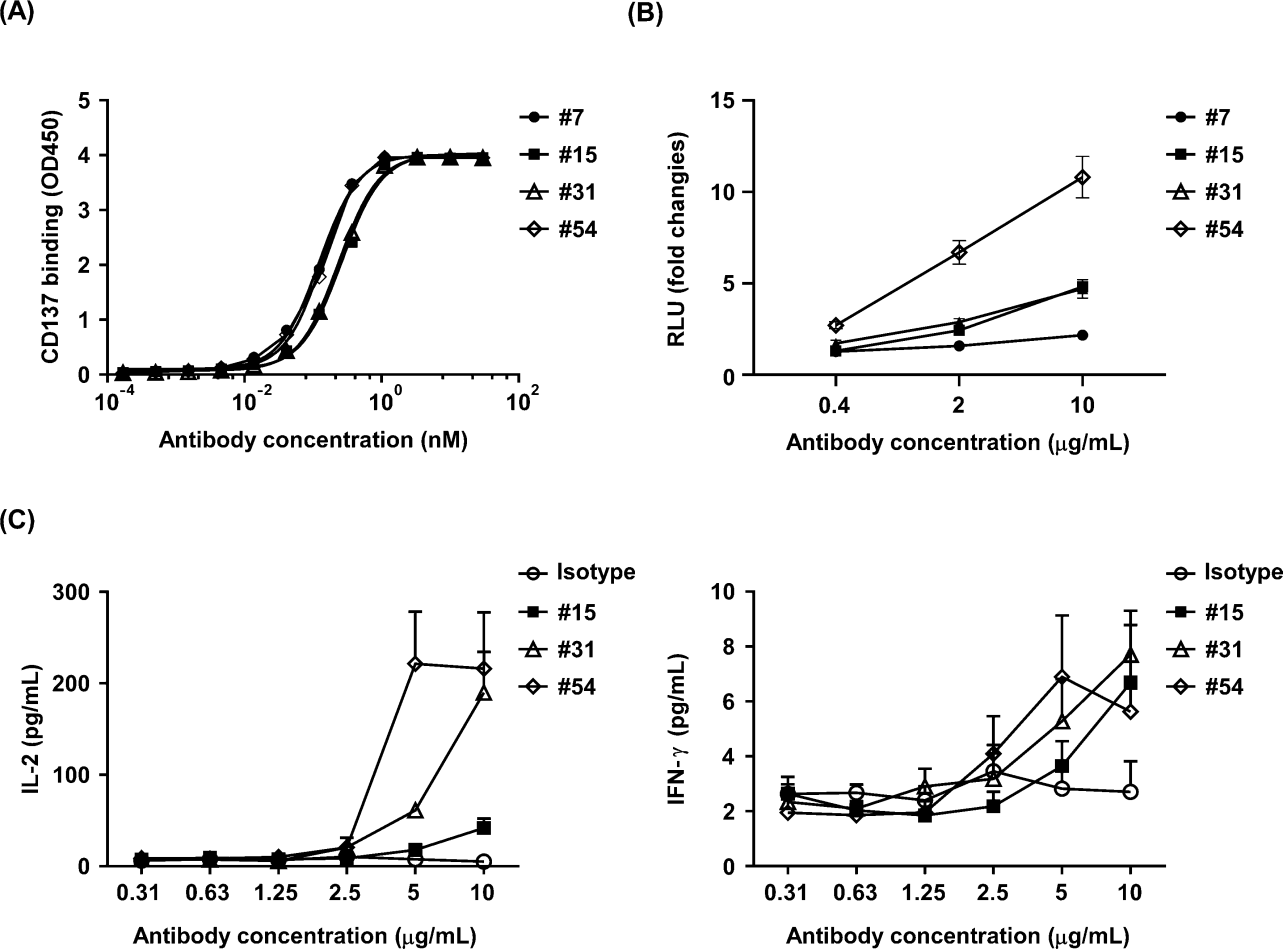
Screening of potent CD137 binders through OmniMab phagemid library. **A** Top four high-affinity binders that bind to CD137. The binding affinity of four CD137 antibody candidates to recombinant CD137-ECD-Fc was determined using ELISA. Clone #7 and clone #54 showed the best binding affinity to CD137-ECD-Fc **B** Agonism analysis of the top four CD137 antibody candidates using a CD137/NF-κB reporter assay. Clone #54 showed the stronger CD137 agonist activity. **C** Induction of IL-2 and IFN-γ secretion by the top four CD137 antibody candidates. The secretion of IL-2 and IFN-γ in the culture supernatant was measured by ELISA. Clone #54 exhibited the best biological activity in inducing secretion of the cytokines IL-2 and IFN-γ

On the other hand, potential CD137 binder candidates were also screened using the OmniMab phagemid library and reformatted into IgG4. Figure 2A shows the top four clones with high binding affinity to CD137. Among them, clone #7 (EC_50_ = 0.12 nM) and clone #54 (EC_50_ = 0.13 nM) had the best binding affinity against CD137, followed by clone #31 (EC_50_ = 0.24 nM) and clone #15 (EC_50_ = 0.26 nM). The CD137 agonism of the four binders was then analyzed using CD137/NF-κB reporter assay (Fig. 2B). Notably, clone #54 had the stronger CD137 agonist activity than other three clones and activated the CD137-dependent NF-κB reporter in a dose-dependent manner. In contrast, clone #7 did not activate the NF-κB reporter at all, although it did show good binding affinity for CD137. Therefore, the effect of the remaining three CD137 binders on T cell activation was further evaluated. As shown in (Fig. 2C), clone #54 had the best bioactivity in promoting the secretion of cytokine IL-2 and IFN-γ, indicating that clone #54 is the potent agonist for CD137.

Afterwards, the two potent lead binders for PD-L1 (clone #6) and CD137 (clone #54) were chosen to generate bispecific antibody AP203 due to their high binding affinity, specific blocking activity and excellent enhancement of T cell responses.

### AP203 exhibits high binding properties to PD-L1 and CD137

**Fig. 3:**
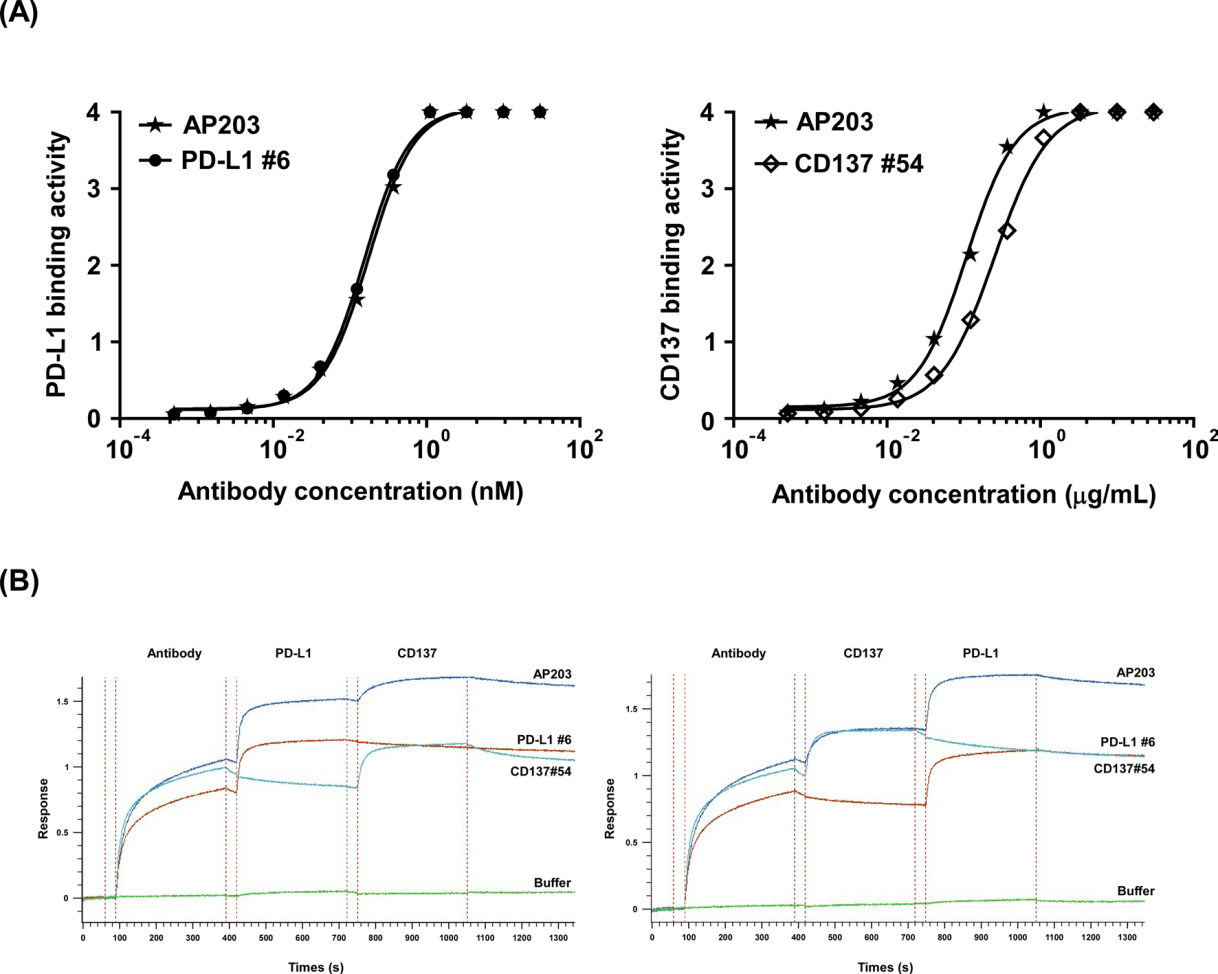
High binding affinity of bispecific antibody AP203 to PD-L1 and CD137. **A** Comparison of the binding affinities of bispecific antibody AP203 and its parental antibody PD-L1 clone #6 and CD137 clone #54 to recombinant human PD-L1 (left panel) and CD137 (right panel), respectively. **B** Simultaneous binding of bispecific antibody AP203 to human PD-L1 and CD137 were measured by bio-layer interferometry

To understand whether the constructed bispecific antibody AP203 impair the native antibody binding ability, the binding affinity of AP203 to PD-L1 and CD137 were assessed and compared with the two parental monomeric forms of the binder (#6 and #54). As shown in Fig. 3A, the binding affinity of AP203 to PD-L1 and CD137 were 0.18 nM and 0.11 nM, respectively, which were similar to that of parental monospecific PD-L1 binder (0.16 nM) and CD137 binder (0.25 nM). In addition, the affinity of AP203 to PD-L1 and CD137 was further assessed by bio-layer interferometry. The results showed that AP203 can simultaneously bind to PD-L1 and CD137 (Fig. 3B), indicating that constructed bispecific antibody AP203 exhibits high binding affinity for PD-L1 and CD137.

### AP203 potently activates T cells, enhances memory recall responses, and overcomes Treg-mediated immunosuppression

**Fig. 4:**
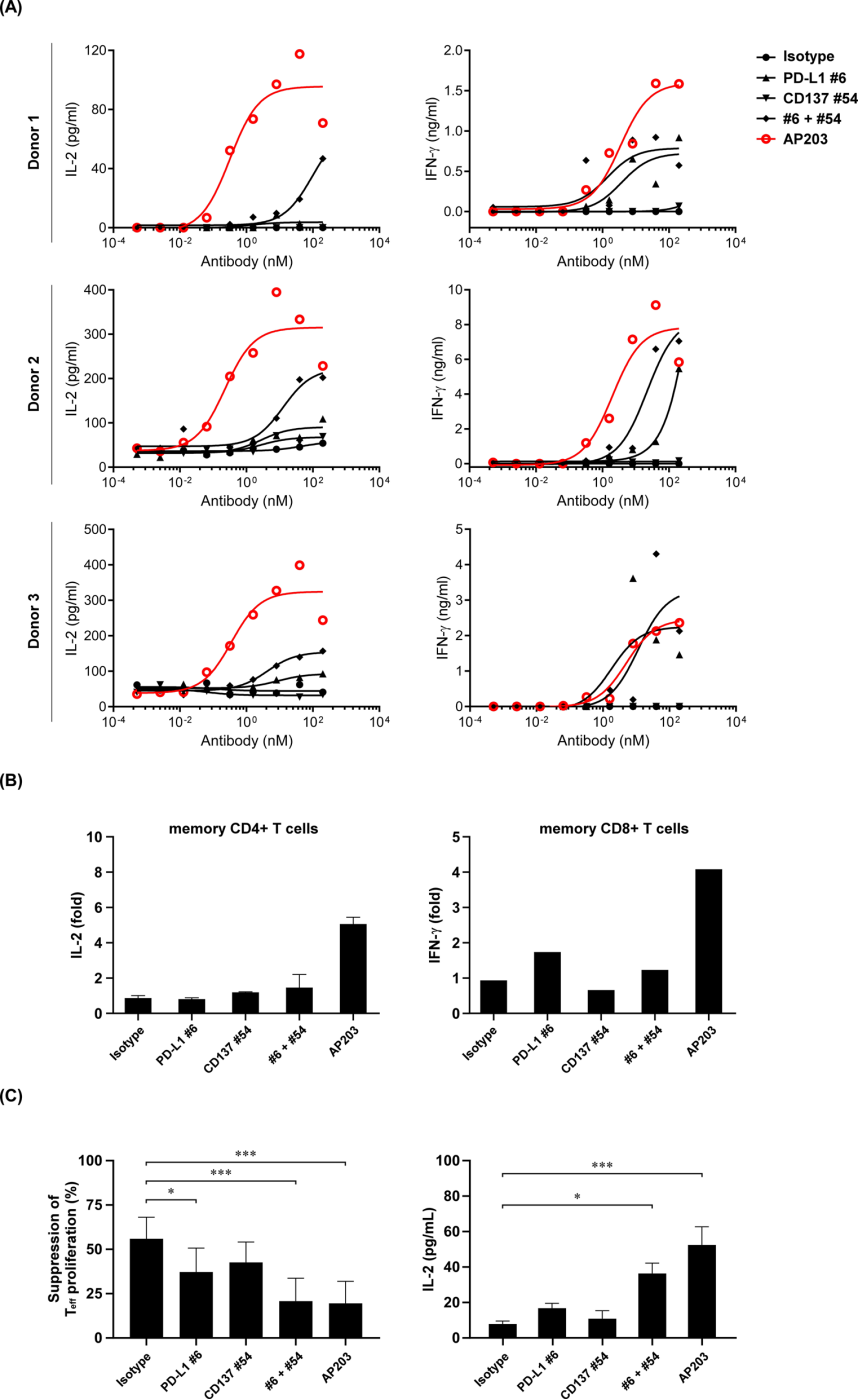
Comparison of AP203 and its parental antibody in T cell activation, enhanced memory recall response and overcoming Treg-mediated immunosuppression. **A** Effect of AP203 in the activation of primary CD4 + T cells in the allogeneic MLR. ELISA was used to measure the secretion of IL-2 and IFN-γ from three healthy donors (DP). AP203 showed superior stimulatory activity in CD4 + T cell activation compared to parental antibody alone or in combination. **B** Memory T cell recall assay measuring IL-2 and IFN-γ produced by human CD4 + memory T cells and CD8 + memory T cells, respectively. The secretion of IL-2 and IFN-γ in the culture supernatant was measured by ELISA. AP203 exerted superior CD4 + and CD8 + memory T cell recall response than its parental antibody alone or in combination. **C** Effect of AP203 in overcoming Treg-mediated suppression of effector T cell proliferation and IL-2 production by CD4 + T cells in the allogeneic MLR. AP203 exhibited superior bioactivity in overcoming Treg-mediated immunosuppression than its parental antibody alone or in combination

The immune stimulatory activity of AP203 was measured by allogenic MLR and compared to that of its parent antibodies. Treatment with PD-L1 #6 and CD137 #54 alone induced little or no secretion of IL-2, whereas combined treatment PD-L1 #6 and CD137 #54 induced more IL-2 secretion (Fig. 4A, left panel). Notably, AP203 treatment further enhanced IL-2 secretion in all three donor pairs in a dose-dependent manner. Similarly, AP203 was shown to be superior to PD-L1 #6 and CD137 #54 alone or in combination in promoting the secretion of IFN-γ (Fig. 4A, right panel).

Next, memory T cell recall assay was used to examine the effect of AP203 in antigen-dependent T cell activation. As shown in Fig. 4B, AP203 treatment enhanced the secretion of IL-2 and IFN-γ, whereas PD-L1 #6 and CD137 #54 antibody alone or in combination induced lower secretion of IL-2 and IFN-γ. In summary, AP203 had superior bioactivity to boost the activation of human primary T cells.

In the tumor microenvironment, infiltrated regulatory T cells (Treg) are known to promote tumor development and progression by suppressing T cell-mediated antitumor immunity [40]. In addition, Treg plays a crucial role in regulating effector CD4 + T cell proliferation, development and function [8, 41]. Therefore, we next examined whether AP203 could overcome Treg-mediated T cell proliferation and immunosuppression. As shown in the left panel of Fig. 4C, Treg-mediated suppression of effector T cell proliferation was significantly reduced after the addition of PD-L1 #6 antibody (P < 0.05). In contrast, the addition of CD137 #54 antibody did not reduce suppressed proliferation of effector T cell caused by Treg cells and had no boost effect when combined with PD-L1 #6 antibody (P > 0.05). Notably, bispecific antibody AP203 significantly reduced Treg-mediated suppression of effector T cell proliferation (P < 0.001). Likewise, AP203 was superior to PD-L1 #6 and CD137 #54 antibodies alone or in combination in restoring Treg-mediated suppression of IL-2 secretion (Fig. 4C, right panel). Collectively, AP203 was able to effectively overcome Treg-mediated immunosuppression.

### AP203-mediated T cell activation is dependent on CD137 cross-linked to PD-L1

**Fig. 5:**
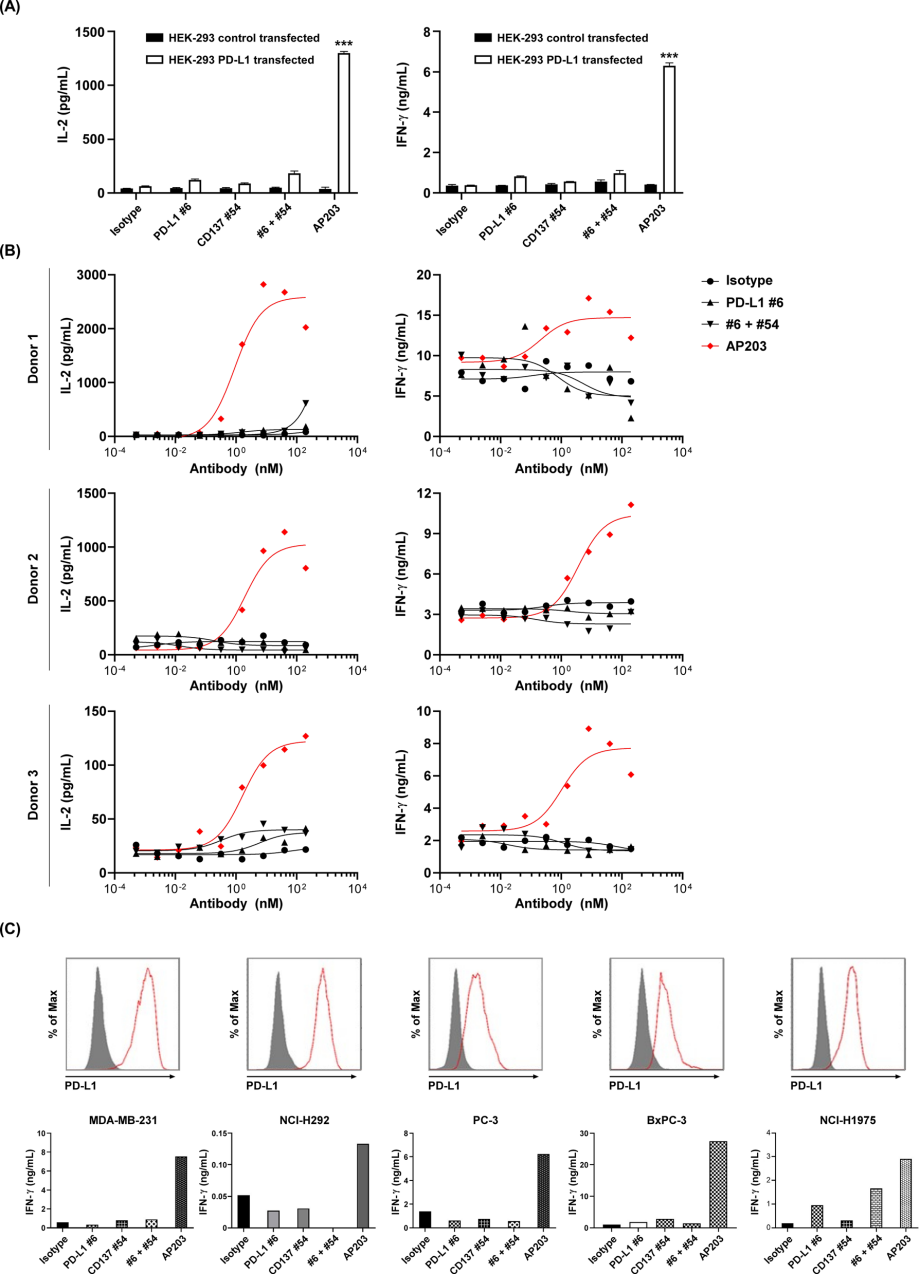
AP203-mediated T cell activation is dependent on the PD-L1 and CD137 cross-linking. **A** AP203 promoted T cell activation in the presence of PD-L1-expressing HEK293 cells. T cells cocultured with HEK293 cells with/without PD-L1 overexpression were treated with control antibody, AP203 or its parental antibody alone or in combination for 3 days. The secretion of IL-2 and IFN-γ in the culture supernatant was measured by ELISA. **B** AP203 promoted T cell activation in a dose-dependent manner when T cells were cocultured with PD-L1 expressing HEK293 cells. The secretion of IL-2 and IFN-γ by T cells from three different donors was measured by ELISA. **C** AP203 promoted T cell activation when cocultured with various cancer cells expressing PD-L1. Upper panel: surface expression of PD-L1 on MDA-MB-231 (breast cancer cells), NCI-H292 (lung mucoepidermoid carcinoma cells), PC-3 (prostate cancer cells), BxPC-3 (prostate cancer cells), and NCI-H1975 (non-small cell lung cancer cells). PD-L1 expression on the cell surface was assessed by flow cytometry. Lower panel: IL-2 and IFN-γ secretion levels were measured in human CD8 + T cells co-cultured PD-L1-expressing cancer cells after treated with isotype control antibody or AP203 or its parental antibody alone or in combination

It has been known that targeting CD137 costimulatory molecule is responsible for the activation of T cells and subsequent transcriptional activation of IL-2 and IFN-γ [42, 43], as also evidenced in Fig. 2. Since AP203 is structurally designed to provide cross-linking between PD-L1^+^ cells and CD137^+^ cells, the bridging effect of AP203 in enhancing T cell activation was first confirmed by using PD-L1-expressing HEK293 cells and CD137-expressing T cells. As shown in Fig. 5A, PD-L1 #6 and CD137 #54 antibodies alone or in combination did not enhance IL-2 and IFN-γ secretion levels (P > 0.05). Notably, AP203 significantly enhanced IL-2 and IFN-γ secretion in the presence of PD-L1-expressing HEK293 cells (P < 0.001). In contrast, AP203-induced IL-2 and IFN-γ secretion was not observed when co-incubated with wild-type HEK293 cells, suggesting that AP203-induced T cells activation only when T cells were co-incubated with PD-L1 positive HEK293 cells. Furthermore, dose-dependent IL-2 and IFN-γ secretion from activated T cells following AP203 treatment was observed in all three independent donors (Fig. 5B).

Given the excellent results of AP203 in promoting T cell activation above, we next confirm whether AP203 retains its superior effects in promoting T activation under various PD-L1-expressing tumor cells, a model that mimics the tumor microenvironment. MDA-MB-231, NCI-H292, PC-3, BxPC-3, and NCI-H1975 were all PD-L1-positive tumor cells (Fig. 5C, upper panel). Consistent with the results from PD-L1-expressing HEK293 cells, AP203 induced significantly higher IL-2 and IFN-γ secretion than PD-L1 #6 and CD137 #54 antibodies alone or in combination (Fig. 5C, bottom panel).

## Anti-tumor efficacy of bispecific antibody AP203 in humanized mouse tumor models of pancreatic and colon cancer

**Fig. 6:**
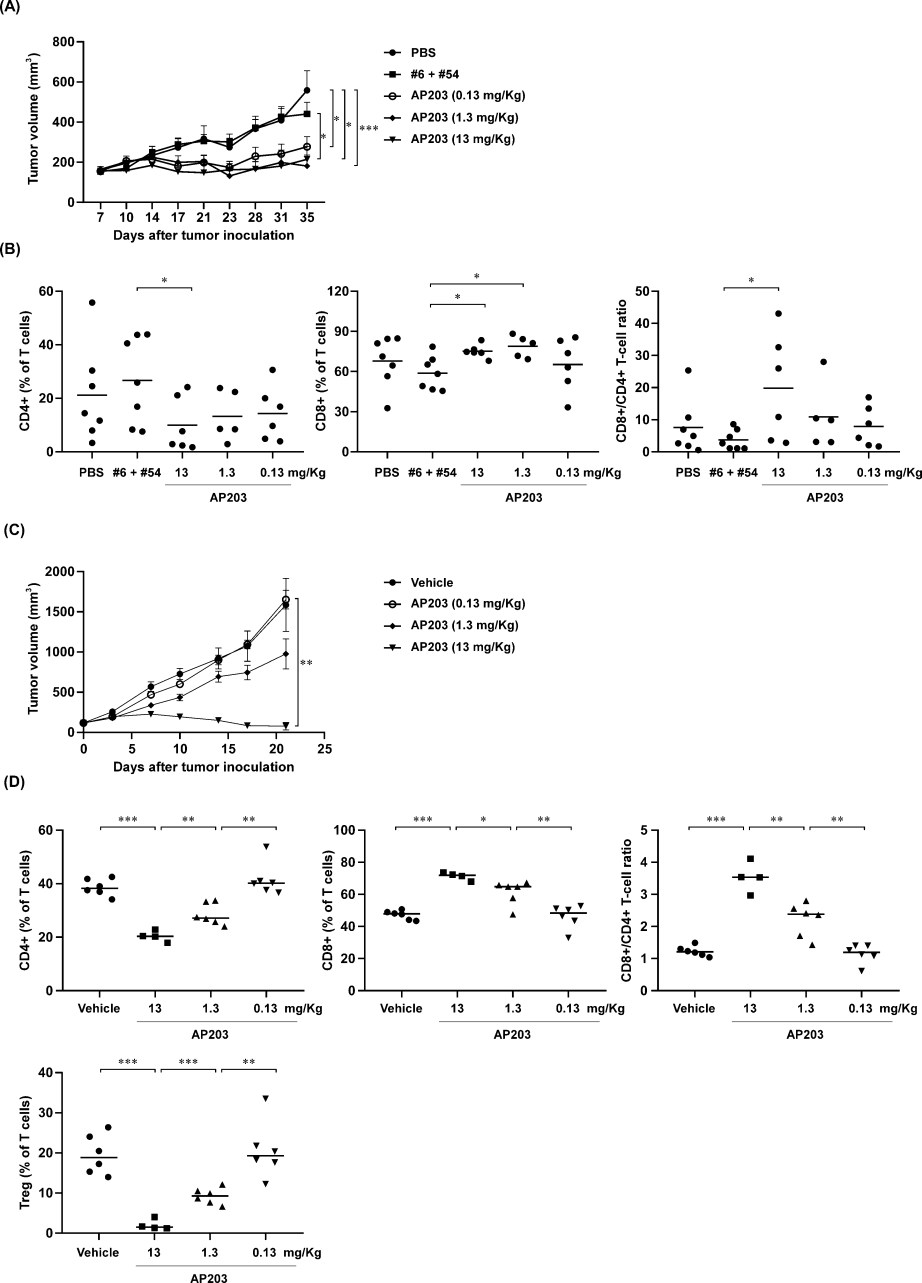
AP203 exerts potent antitumor activity in two humanized mouse models. **A** Anti-tumor efficacy of AP203 in the immunodeficient SCID/Beige mice xenografting with BxPC-3 pancreatic tumors reconstituted with human immune cells. Mice bearing BxPC-3 xenograft tumors were injected intraperitoneally with PBS control (10 mg/kg) or AP203 (0.13, 1.3, and 13 mg/kg) or its parental antibodies (each 10 mg/kg) in combination for twice a week. The tumor volumes were measured and recorded twice a week and presented as mean ± SEM. Differences were found to be statistically significant at * < 0.05 and *** < 0.001. **B** Analysis of infiltrating human lymphocyte population in BxPC-3 tumor tissues. At day 35, BxPC-3 tumor tissues were collected for analysis of the tumor-infiltrating human lymphocytes by flow cytometry. **C** Anti-tumor efficacy of AP203 in PD-1/PD-L1/CD137 triple knock-in mice bearing MC38 colon cancer cells. MC38 tumor-bearing mice were injected intraperitoneally with PBS control or AP203 or its parental antibodies in combination for twice a week. The tumor volumes were measured and recorded twice a week and presented as mean ± SEM. Differences were found to be statistically significant at ** < 0.01. **D** Altered TILs populations in MC38 subcutaneous colon tumors following treatment with bispecific antibody AP203. Flow cytometry analysis was used to determine and quantify different immune cells (% of either live or CD45 + cells)

Next, we examined the in vivo anti-tumor efficacy of AP203 in immunodeficient SCID/Beige mice xenografting with BxPC-3 pancreatic tumor cells reconstituted with human immune cells. As shown in Fig. 6A, AP203 treatment significantly inhibited tumor growth compared to PBS control groups and the combination of PD-L1 #6 and CD137 #54 antibodies group (P < 0.05). Notably, good antitumor efficacy was also observed when treated with lowest dose of AP203 (0.13 mg/Kg). In contrast, the combination of PD-L1 #6 and CD137 #54 antibodies only slightly inhibited tumor growth on day 35, with no statistical significance compared to the PBS control (P > 0.05). To understand the impact of AP203 in the tumor immune microenvironment, tumor infiltrating lymphocytes (TILs) were isolated from tumor tissues and analyzed by flow cytometry. There were no significant differences in the percentage of CD4 + , CD8 + T cells and the CD8 + /CD4 + ratio between the PBS control and the combination of PD-L1 #6 and CD137 #54 antibodies (Fig. 6B, P > 0.05). In contrast, AP203 treatment significantly increased the percentage of CD8 + T cells and decreased the percentage of CD4 + T cells (P < 0.05), resulting in a dose-dependent increase in the CD8 + /CD4 + ratio. These results suggest that AP203 treatment affect tumor microenvironment. The anti-tumor efficacy of AP203 was further confirmed using human PD-1/PD-L1/CD137 triple knock-in mice bearing human PD-L1-knock-in MC38 colon cancer cells. As shown in Fig. 6C, AP203-treated mice showed a significantly reduction in tumor volume relative to the PBS control (P < 0.001). Likewise, infiltrating TILs populations in the tumor tissues were marked changed after AP203 treatment. The CD8 + /CD4 + T cell ratio was significantly increased after AP203 treatment (P < 0.001), which was attributed to a marked increase in infiltrating CD8 + T cells and a marked decrease in infiltrating CD4 + T cells (Fig. 6D). Notably, the population of Treg cells were significantly reduced in tumors treated with AP203 (P < 0.001). Collectively, the in vivo findings clearly revealed the good anti-tumor efficacy of AP203, which may be mediated by modulating tumor immune microenvironment, including expansion of cytotoxic CD8 + T cells and suppression of Treg cells.

### AP203 does not induce human PBMCs to produce perimammary cytokines

**Fig. 7:**
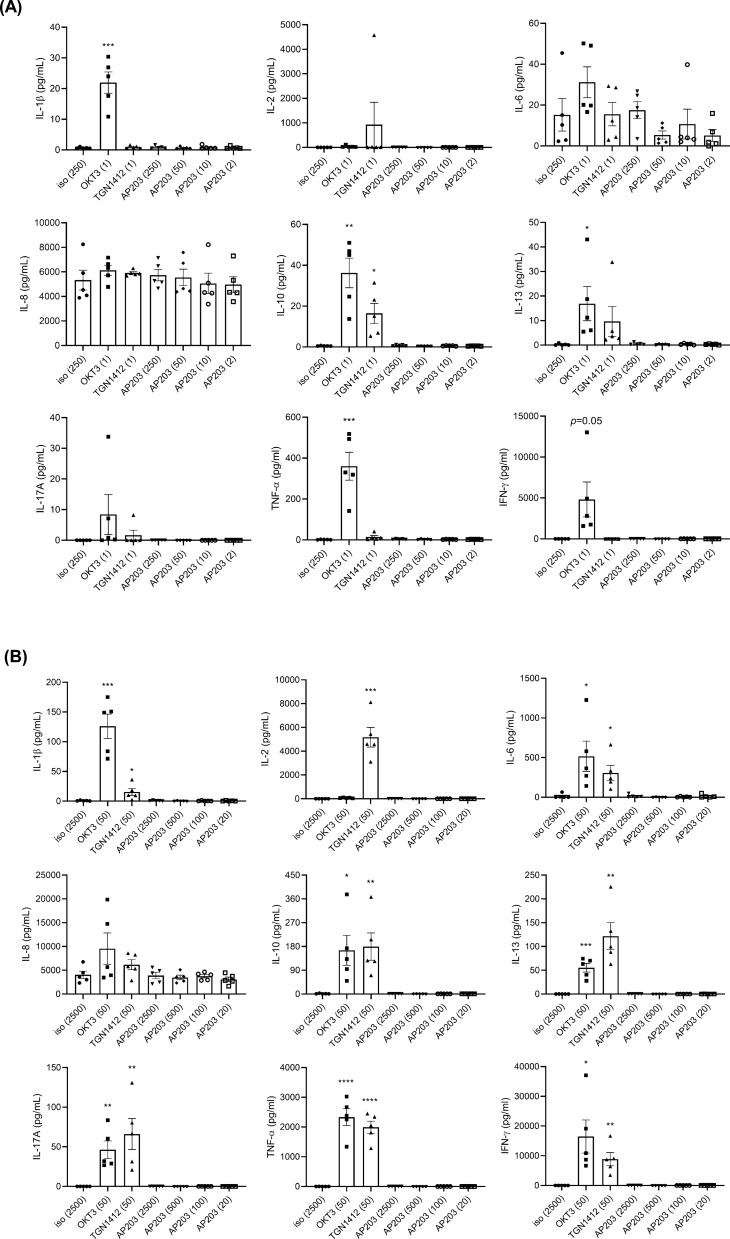
Incapacity of bispecific antibody AP203 to induce the production of proinflammatory cytokines. PBMCs from healthy donors were treated with isotype antibody (negative control), OKT3 (positive control), TGN1412 (positive control), and soluble **A** or immobilized **B** bispecific antibody AP230 for 24 h. The levels of the secreted cytokines i IL-1β, IL-2, IL-6, IL-8, IL-10, IL-13, IL-17A, TNF-α, and IFN-γ in the culture supernatant were measured by ProcartaPlex Immunoassays. Even with high-dose AP230 treatment, inflammatory cytokines were not significantly elevated, indicating the safety of AP203

Since cytokine release syndrome would be the vital adverse effect in the use of immunoregulatory antibodies, we next examined whether AP203 treatment induces the release of various cytokines from human immune cells. Human PBMCs from 5 healthy donors were isolated and stimulated with antibodies in the soluble form or the immobilized form, which triggers stronger cytokine release upon antibody crosslinking [44]. Cytokine release levels including IL-1β, IL-2, IL-6, IL-8, IL-10, IL-13, IL-17A, TNF-α, and IFN-γ were measured by ProcartaPlex Immunoassays. The results showed that OKT3 or TGN1412 treatment significantly induced the release of various cytokines. However, treatment with extremely high concentrations of soluble AP203 (up to 250 μg/ml) or even with immobilized AP203 (up to 2500 ng/well) did not increase proinflammatory cytokine production (Fig. 7).

## Discussion

Despite recent advances in immune checkpoint blockade in the landscape of cancer treatment, unsatisfactory response rate, resistance and immune-related side effects have greatly limited clinical efficacy of monoclonal antibody strategies and turned to the combination of immune checkpoint blockade therapy [45–47]. Considering the advantage of bispecific antibodies in redirecting immune effector cells to the vicinity of tumor cells, a bispecific antibody AP203 was developed to block tumor PD-L1-medicated immunosuppression and simultaneously activate redirected effector T cells. The in vitro results revealed that bispecific antibody AP203 not only exhibited superior agonistic effects on T cell activation than parental antibodies alone or in combination, but also more effectively overcome Treg-mediated immunosuppression. Moreover, AP203 was almost completely incapable of promoting cytokine production by human PBMCs, which may more or less eliminate concerns about systemic cytokine release in cancer immunotherapy. Furthermore, in vivo tumor models not only confirmed the potent anti-tumor efficacy of AP203, but also revealed that AP203 treatment can modulate the immunosuppressive tumor microenvironment, with evidence of cytotoxic CD8 + T cell expansion and Treg cell suppression.

For the treatment of solid tumor, suppressive tumor microenvironment is the major hindering factor because it effectively impedes the activation of infiltrating T cells [37, 48]. The PD-1/PD-L1 pathway and Tregs infiltration are two essential factors for tumor cells to escape immune surveillance and suppress the anti-tumor immune response [49]. Therefore, targeting PD-1 or PD-L1 is the primary goal of constructing bispecific antibody AP203. However, some studies reported the co-expression of PD-1 and PD-L1 on the surface of antigen presenting cells or tumor cells [50–53]. The PD-1 and PD-L1 on the surface of tumor cells can interact with each other in cis. Importantly, PD-1 antibody may block the cis PD-1/PD-L1 interaction on tumor cells to release PD-L1, which may tend to bind PD-1 on T cells and then suppress T cell activation [54]. Taken this into consideration, the important feature of the bispecific antibody AP203 was designed to target PD-L1 rather than PD-1.

In addition to disrupting the canonical PD-1/PD-L1 inhibitory signals, simultaneously triggering the activation of these T cells redirected to the vicinity of cancer cells is the advantage of the bispecific antibody AP203. In cancer immunotherapy, in addition to T cell receptor (TCR) ligation, fully T cell activation requires further nonspecific co-stimulatory signals, such as the CD137 co-stimulatory molecule [55]. Currently, urelumab (Bristol-Myers Squibb) and utomilumab (Pfizer) are two CD137 agonist monoclonal antibody. Despite the promising therapeutic efficacy of urelumab, dose-limiting hepatotoxicity quickly emerged in some cases, temporarily putting the development program on hold [56]. In contrast, single agent utomilumab did not show dose-limiting hepatotoxicity but was less effective relative to urelumab [57], and was considered to achieve greater clinical benefit in combination with other monoclonal antibodies [58, 59]. In addition to the combined application of multiple antibodies, bispecific antibodies encompassing both activities in a single moiety may be a better strategy [6]. Several studies also reported the potential of engineered bispecific antibody that combine PD-L1 and CD137 agonists for anti-tumor immunity [60, 61]. In fact, the strength of CD137 agonism is known to depend on the different antibody binding epitopes towards the CD137, as evidenced in this study that a panel of CD137 clones did exhibit different binding affinity and agonist profiles. Benefiting from the high binding affinity of AP203 to PD-L1 and its potent agonism to CD137, AP203 can crosslink PD-L1 on tumor cells and CD137 on T cells to stabilize the immune synapses and further promote the activation of T cells. As evidenced by the results of this study, our bispecific antibody AP203 indeed potently triggered T cell activation, increased infiltration of CD8 + T cells into tumors, and promoted the production of effector cytokines.

On the other hand, in order to achieve a balance between therapeutic efficacy and immune-related adverse events, two modifications were made to the Fc region of AP203 to avoid potential antibody dependent cellular cytotoxicity (ADCC) and complement-dependent cytotoxicity (CDC). The N297A mutation was created to prevent Fcγ receptor binding by diminishing N-glycosylation, while the K322A mutation was generated to reduce antibody-induced allodynia by blocking complement activation [62, 63]. In addition, a recent study by Wang et al. demonstrated that mutational silencing of the Fc domain of bispecific antibodies is necessary to drive T cell trafficking to solid tumors for improving antitumor potency [64]. Effective as expected, our PBMC-based T cell functional assessment supported that AP203 was almost completely incapable of promoting cytokine production. In addition, CD8 + tumor-infiltrating T cells were significantly increased, and infiltrating Treg cells were significantly decreased, increasing our confidence in subsequent clinical trials of AP203.

## Conclusions

AP203 is a fully human bispecific antibody with high binding affinity to PD-L1 and CD137, and exerts potent antitumor activity without toxicity. The mechanism of action of AP203 relies on blocking the PD-1/PD-L1 inhibitory signals through the PD-L1 arm, activating effector T cells through the CD137 arm, and redirecting T cells through cross-linking, as well as overcoming Treg-mediated immunosuppression. The promising preclinical results strongly support the translational application of AP203 in subsequent clinical trials for the treatment of locally advanced or metastatic solid tumors (APT-CUBE, ClinicalTrials.gov Identifier: NCT05473156).

### Supplementary Information

**Additional file 1: Figure S1.**Schematic diagram of the structure of bispecific antibody AP203. The AP203 construct was designed as two connected units. One unit contains a full antibody backbone with high specific binding affinity to PD-L1, and the other consists of two basic single-chain fragments with variable domains with high specific binding affinity to CD137.Purified bispecific antibody AP203. After purification by Protein A affinity chromatography, purified AP203 under reducing and non-reducing conditions was examined by SDS-PAGE, and the purity was examined by SEC-HPLC. Abbreviation: CH, heavy chain constant region; CL, light chain constant region; VH, heavy chain variable region; VL, light chain variable region; SEC-HPLC, size-exclusion chromatography-high performance liquid chromatography.

## Data Availability

All data analyzed and used in this study are available from the corresponding author upon reasonable request.

## Abbreviations

PD-1: Programmed cell death-1
PD-L1: Programmed cell death ligand-1
ELISA: Enzyme-linked immunosorbent assay
BLI: Biolayer interferometry
MLR: Mixed lymphocyte reaction
PBMCs: Peripheral blood mononuclear cells
Treg cells: Regulatory T cells
irAEs: Immune-related adverse events
DCs: Dendritic cells
NK cells: Natural killer cells
TILs: Tumor infiltrating lymphocytes
HEL: Hen Egg Lysozyme
FACS: Fluorescence-activated cell sorting
scFv: Single-chain variable fragment
SEC-HPLC: Size-exclusion chromatography-high performance liquid chromatography
