# Correction: A bispecific antibody AP203 targeting PD-L1 and CD137 exerts potent antitumor activity without toxicity

**DOI:** 10.1186/s12967-023-04410-1

**Published:** 2023-08-16

**Authors:** Po-Lin Huang, Hung-Tsai Kan, Ching-Hsuan Hsu, Hsin-Ta Hsieh, Wan-Chien Cheng, Ren-Yeong Huang, Jhong-Jhe You

**Affiliations:** 1 AP Biosciences, Inc., 17F., No. 3, Yuanqu St., Nangang Dist., Taipei, 115603 Taiwan; 2 Department of Periodontology, School of Dentistry, Tri-Service General Hospital and National Defense Medical Center, Taipei, Taiwan

**Correction: Journal of Translational Medicine (2023) 21:346** 10.1186/s12967-023-04193-5

Following publication of the original article [1], we have been notified that the published article text contains incorrect wording. It should be as follows:

Humanized in Results and Conclusions’ section should be replaced with fully human.
