# Supplementary figures and images for: A bispecific antibody AP203 targeting PD-L1 and CD137 exerts potent antitumor activity without toxicity
Source: J Transl Med. 2023 May 25;21:346. doi: 10.1186/s12967-023-04193-5 (PMC10210478; doi:10.1186/s12967-023-04193-5)

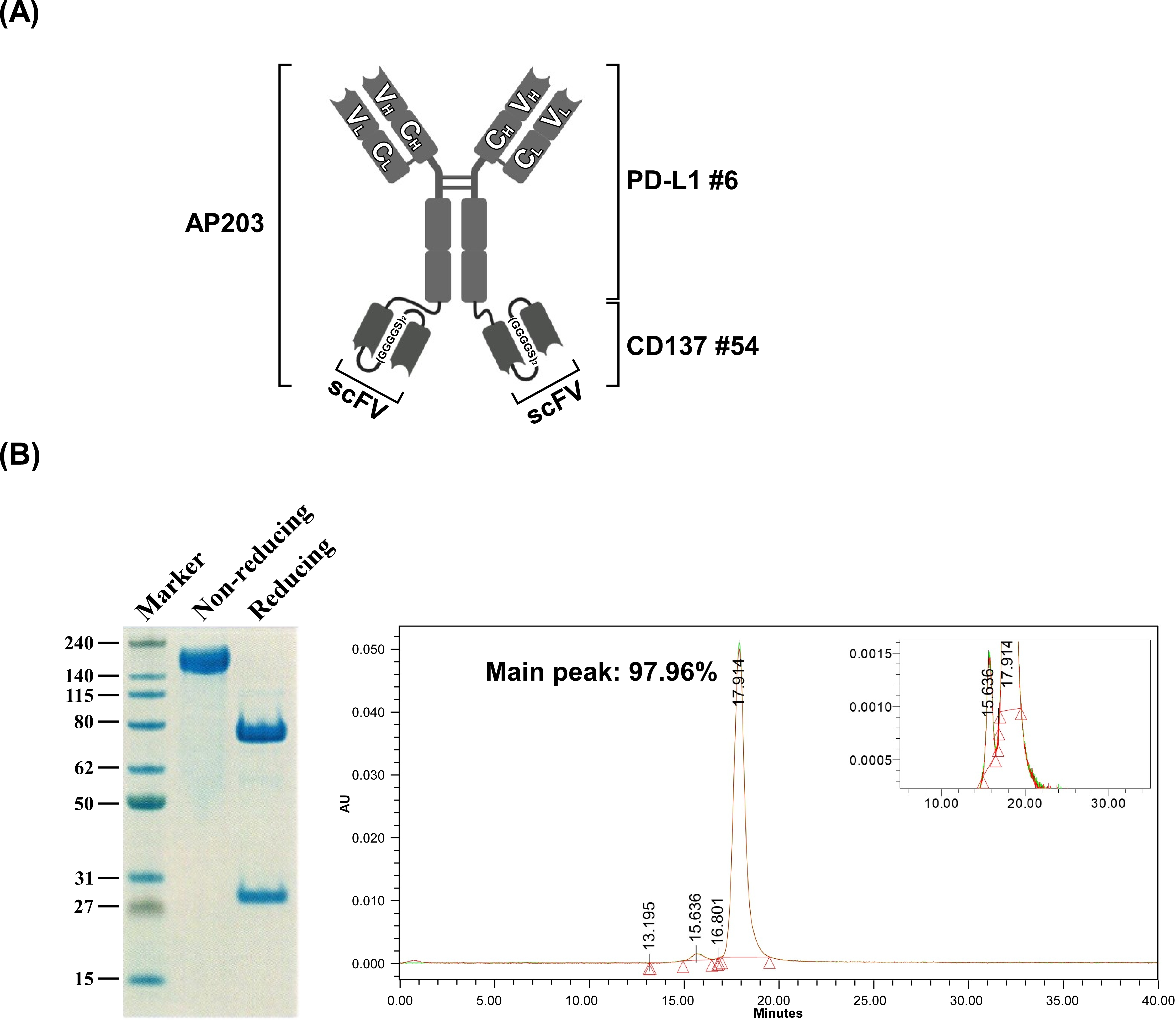

Supplement: Supplementary file 1 — Additional file 1: Figure S1.Schematic diagram of the structure of bispecific antibody AP203. The AP203 construct was designed as two connected units. One unit contains a full antibody backbone with high specific binding affinity to PD-L1, and the other consists of two basic single-chain fragments with variable domains with high specific binding affinity to CD137.Purified bispecific antibody AP203. After purification by Protein A affinity chromatography, purified AP203 under reducing and non-reducing conditions was examined by SDS-PAGE, and the purity was examined by SEC-HPLC. Abbreviation: CH, heavy chain constant region; CL, light chain constant region; VH, heavy chain variable region; VL, light chain variable region; SEC-HPLC, size-exclusion chromatography-high performance liquid chromatography. [file 12967_2023_4193_MOESM1_ESM.jpg]
